# Case report: two novel *PPARG* pathogenic variants associated with type 3 familial partial lipodystrophy in Brazil

**DOI:** 10.1186/s13098-024-01387-9

**Published:** 2024-07-01

**Authors:** Monique Alvares da Silva, Reivla Marques Vasconcelos Soares, Antônio Fernandes de Oliveira Filho, Leonardo René Santos Campos, Josivan Gomes de Lima, Julliane Tamara Araújo de Melo Campos

**Affiliations:** 1 Laboratório de Biologia Molecular e Genômica, Departamento de Biologia Celular e Genética, Centro de Biociências, Universidade Federal do Rio Grande do Norte - UFRN, Campus Universitário, Lagoa Nova, Natal, RN 59072-970 Brazil; 2 Departamento de Medicina Clínica, Hospital Universitário Onofre Lopes, Universidade Federal do Rio Grande do Norte - UFRN, Natal, RN Brazil; 3 Núcleo de Tecnologia em Saúde (NUTES), Universidade Estadual da Paraíba - UEPB, Campina Grande-PB, Brazil; 4 Bioinformatics Multidisciplinary Environment, Universidade Federal do Rio Grande do Norte - UFRN, Natal, RN Brazil

**Keywords:** Familial partial lipodystrophy, PPARγ, Adipose tissue

## Abstract

**Introduction and aim:**

Type 3 Familial Partial Lipodystrophy (FPLD3) is a rare metabolic disease related to pathogenic *PPARG* gene variants. FPLD3 is characterized by a loss of fatty tissue in the upper and lower limbs, hips, and face. FPLD3 pathophysiology is usually associated with metabolic comorbidities such as type 2 diabetes, insulin resistance, hypertriglyceridemia, and liver dysfunction. Here, we clinically and molecularly characterized FPLD3 patients harboring novel *PPARG* pathogenic variants.

**Materials and methods:**

Lipodystrophy-suspected patients were recruited by clinicians from an Endocrinology Reference Center. Clinical evaluation was performed, biological samples were collected for biochemical analysis, and DNA sequencing was performed to define the pathogenic variants associated with the lipodystrophic phenotype found in our clinically diagnosed FPLD subjects. Bioinformatics predictions were conducted to characterize the novel mutated PPARγ proteins.

**Results:**

We clinically described FPLD patients harboring two novel heterozygous *PPARG* variants in Brazil. Case 1 had the c.533T > C variant, which promotes the substitution of leucine to proline in position 178 (p.Leu178Pro), and cases 2 and 3 had the c.641 C > T variant, which results in the substitution of proline to leucine in the position 214 (p.Pro214Leu) at the PPARγ2 protein. These variants result in substantial conformational changes in the PPARγ2 protein.

**Conclusion:**

Two novel *PPARG* pathogenic variants related to FPLD3 were identified in a Brazilian FPLD cohort. These data will provide new epidemiologic data concerning FPLD3 and help understand the genotype-phenotype relationships related to the *PPARG* gene.

**Supplementary Information:**

The online version contains supplementary material available at 10.1186/s13098-024-01387-9.

## Introduction

Congenital lipodystrophies are rare heterogeneous syndromes characterized by a partial or generalized loss of subcutaneous white adipose tissue (sWAT), resulting in Familial Partial Lipodystrophy (FPLD) or Congenital Generalized Lipodystrophy (CGL). FPLD presents an estimated prevalence of 1.67 cases per million people. The prevalence of partial and generalized lipodystrophies is estimated at 2.63 cases per million [1]. CGL has a worldwide prevalence of 1 case per 1 million inhabitants. Although a higher number of CGL cases was found in Northeast Brazil, with 32.3 cases per million inhabitants, the Brazilian CGL and FPLD epidemiology is unknown [2]. Therefore, unraveling the clinical and genetic profile of FPLD patients in Brazil is crucial to proposing consistent epidemiological data and guaranteeing proper health care to FPLD Brazilian patients.

Type 3 FPLD (FPLD3) is related mainly to missense variants in the *PPARG* gene, a master regulator of adipogenesis [3]. The loss of sWAT in FPLD3 individuals occurs mainly in the face, legs, and arms during adulthood. The forearms and calves lose more fat than the arms and thighs [4]. Although the loss of sWAT is less severe in FPLD3 compared to type 2 FPLD (FPLD2), the metabolic disturbances found in FPLD3 are more significant. They include a spectrum of clinical features such as hypertension, hypertriglyceridemia, diabetes, insulin resistance, and cardiometabolic complications. Females also may present polycystic ovary syndrome, hirsutism, and excessive production of androgens in the ovaries [5].

The loss of sWAT is a key mechanism for developing metabolic complications in both FPLD2 and FPLD3 types. Besides, the more severe clinical features associated with FPLD3 suggest that the *PPARG* gene may have additional functions besides its role in adipogenesis [6]. Here, we describe two novel *PPARG* heterozygous pathogenic variants affecting the DBD domain and the Hinge region of the PPARγ2 protein. These variants change the conformational PPARγ2 structure and are associated with the clinical features of a new FPLD3 cohort from Brazil.

## Materials and methods

### Subjects and data collection

This study was approved by the Ethical Committee of Hospital Universitário Onofre Lopes (CEP-HUOL) from Universidade Federal do Rio Grande do Norte (UFRN), Natal-RN (Ethical Committee Number 3.655.354). All experiments were performed according to the ethical guidelines of CEP-HUOL and the Declaration of Helsinki (1975). The procedures were clearly explained, and all participants gave their written informed consent. Written informed consent to publish images was obtained for FPLD cases 1, 2, and 3.

We have examined three Brazilian patients suspected of FPLD and conducted a bibliographic search of *PPARG* pathogenic variants affecting the DBD domain and the hinge region using the PubMed, Clinvar, and HGMD databases.

### Anthropometric measurements and dua-energy X-ray absorptiometry (DXA)

BMI was calculated using the weight/height^2^ ratio. All reference values were adopted according to the parameters of the Brazilian Society of Cardiology, Diabetes, and Obesity [7, 8].

Whole body scans by DXA were acquired using Lunar iDXA (GE Healthcare, Bedford, UK), according to the manufacturer’s procedures. Images were analyzed using the iDXA enCORE software (version 14.10.022; GE Healthcare). Fat mass was obtained for the whole body, upper and lower limbs (right and left members), and trunk. The fat mass ratio (FMR) was calculated by dividing trunk fat % by lower limb fat %, a biomarker for FPLD [9]. Baumgartner’s index was calculated by dividing appendicular skeletal muscle by height^2^ (ASM/height^2^) [10]. Fat shadow from the DXA body was obtained according to Meral et al. 2018 [11].

### Biochemical measurements

Biochemical measurements were carried out from plasma collected in a tube containing EDTA according to the protocols proposed by the company Labtest (Labtest Diagnóstica S.A) and using the Labmax Plenno equipment (Labtest, Lagoa Santa, Minas Gerais, Brazil) differing from each other only in the methodology applied to each analyte. The Trinder methodology determined the plasma glucose, triglycerides, and total cholesterol concentrations. The analytes HDL-c, LDL-c, and VLDL-c were carried out using the selective precipitation method, and the non-HDL-c and LDL-c fractions were carried out using the mathematical method of the Friedwald equation. This study did not include controlled fasting of a duration greater than or equal to 8 h for all samples.

Adiponectin was measured with an ELISA kit (Life Technologies Corporation, Invitrogen, USA; catalog number KHP0041). Leptin was determined using an ELISA kit (Life Technologies Corporation, Invitrogen, USA; catalog number KAC228). Both assays were done using the total plasma according to the manufacturing instructions.

### Next-generation sequencing (NGS)

The candidate genes *ABCA1*, *AGPAT2, AKT2, APOA5, APOC2, BSCL2, CAV1, CFTR, CIDEC, CTRC, CYP27A1, GPIHBP1, LIPA, LIPE, LMF1, LMNA, LMNB2, LPL, PLIN1, POLD1, PPARG, PRSS1, PSMB8, SMPD1, SPINK1*, and *ZMPSTE24* were sequenced by next-generation sequencing (NGS) using the HiSeq2000 instrument (Illumina Inc, San Diego, USA). Sequencing was performed for 100 bp, covering each gene’s coding regions and splicing junctions. According to the manufacturer’s protocol, genomic libraries were prepared using Illumina’s Truseq DNA Sample Preparation Kit. The filtered reads were aligned to the human genome (Hg19/GRC38) using the Burrows-Wheeler Aligner (BWA v.0.7.5) [12] Polymerase chain reaction (PCR) duplicates were removed using Picard v1.92 [13] and baseline quality recalibration, indel and SNP realignment, and indel discovery were performed using the Genome Analysis Toolkit (GATK v2.5–2) [12]. Variants were classified according to the Human Genome Variation Society (HGVS) recommendations [14]. The Mutalyzer tool was used to confirm the HGVS nomenclature [15].

### Capillary DNA sequencing

A conventional PCR for the *PPARG* gene regions comprising the novel pathogenic variants was achieved using the Platinum Supermix Kit (Thermofisher Scientific, Waltham, Massachusetts, USA, # 11306152), primers at 0.2 µM targeting the region of the *PPARG* gene (for c.533C > T forward primer: 5′ GGCCAGTATACCTTTCGCTGT3′ and reverse primer: 5′ TGGCAATGGCTTTAGTGTCCA3′ and for c.641C > T forward primer: 5′ GTGGAGGAGGAGGGCTTCTA3′ and reverse primer: 5′ GTGTGTGCATTTGTAGCGCA 3’), and genomic DNA at 100 ng. Since we are describing novel pathogenic variants, we designed the abovementioned primers. The steps of the PCR reaction for the *PPARG* gene were as follows: 95 ◦C for 10 min, 35 denaturation cycles at 95 ◦C for 45 s, annealing at 56 ◦C for 45 s, and extension at 72 ◦C for 1 min. A final step at 72 ◦C for 10 min was performed. The PCR product of 406 bp was purified using the Exosap-It™ PCR Product Cleanup Kit (Thermofisher Scientific, Waltham, Massachusetts, USA, #78,201), according to the manufacturer’s recommendations. Later, the purified amplicons were submitted to a new PCR, using the same *PPARG* primers for conventional PCR, in separated reactions, and the BigDye Terminator V3.1 Cycle Sequencing Kit (Thermofisher Scientific, Waltham, Massachusetts, USA, #4,337,455) was performed according to the manufacturer’s instructions. The purification of the new amplicons was performed using the BigDye Xterminator Purification Kit (Thermofisher Scientific, Waltham, Massachusetts, USA, # 4,376,486) and then analyzed by capillary electrophoresis in the ABI 3500 equipment (Applied Biosystems, Foster City, California, USA).

### Bioinformatics analysis

The mRNA nucleotide sequences of the wild-type PPARγ isoform 2 (PPARγ2; NM_015869.5) were obtained through the National Center for Biotechnology Information (NCBI) database. The nucleotide sequences of the novel PPARγ2 pathogenic variants were obtained by changing the wild-type to the mutated nucleotide in the PPARγ2 mRNA sequence. Then, we used the Translate Tool to obtain the amino acid sequences of the mutated PPARγ2 proteins. Further, as a recommendation of the American College of Medical Genetics and Genomics (ACMG) [16], bioinformatic analysis was performed to determine the pathogenic potential of the novel *PPARG* pathogenic variants using the PolyPhen-2 (Polymorphism Phenotyping v2) [17], the Mutation Taster [18], CADD (Combined Annotation Dependent Depletion) v.1.7 [19] and REVEL (rare exome variant ensemble learner) tools [20]. The Mutation Taster [18] and Mutalyzer tools were also used to confirm the nomenclature of the novel variants according to the main human PPARγ isoforms (1, 2, and 3) (Table S1). T-Coffee [13] was applied to align the wild-type and the mutated PPARγ2 amino acid sequences. Bioinformatics analysis to define the effect of the two novel *PPARG* pathogenic variants on the PPARγ2 protein structure was performed using ChimeraX [21] and AlphaFold Protein [22] tools. These analyses were based on the wild-type PPARγ2 sequence (UniProt P37231; NP_056953.2). The rotamers with the highest prevalence value were considered for the wild-type and mutated PPARγ2 protein structure simulation.

## Results

### Clinical and laboratory evaluation

#### Case 1

**Fig. 1:**
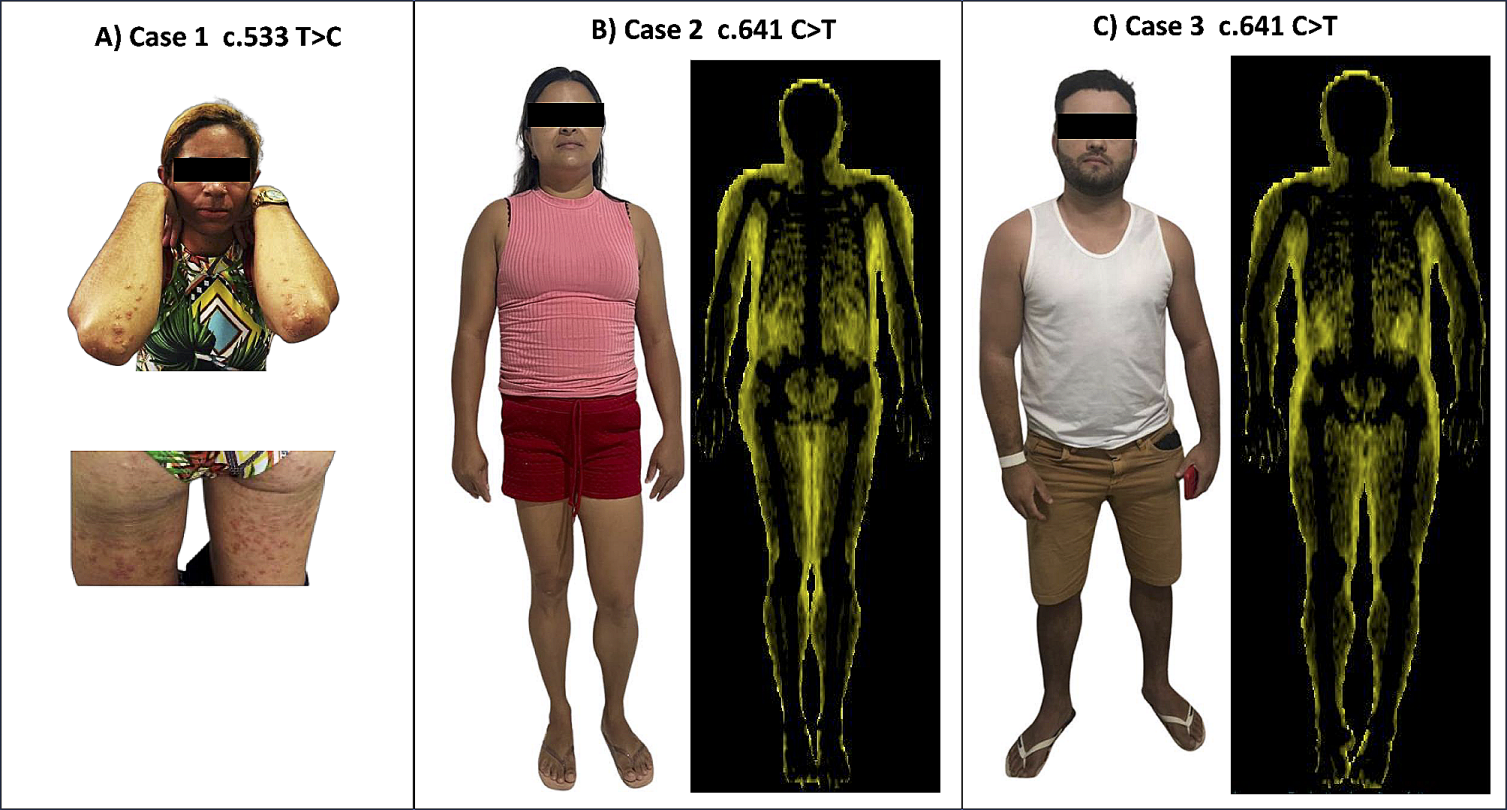
FPLD case images. (**A**) FPLD case 1 (c.533T > C). The patient’s physical examination revealed reduced subcutaneous white adipose tissue (sWAT) in the upper and lower limbs, phlebomegaly, and large numbers of eruptive xanthomas affecting the elbows, buttocks, and dorsal region of the thigh. (**B**) FPLD3 case 2 (c.641 C > T). The patient’s physical examination revealed reduced sWAT in the upper and lower limbs. Fat shadows obtained from DXA scans (right image) showed reduced fat signals in the upper and lower limbs and sWAT accumulation in the neck and axillae. (**C**) FPLD3 case 3 (c.641 C > T). No visible phenotypic changes were observed despite significant dyslipidemia. sWAT reduction was not remarkable. Fat shadows from DXA scans (right image) showed reduced sWAT signals in the upper and lower limbs

A 38-year-old female patient was diagnosed with FPLD at the age of 35 due to difficult-to-control diabetes mellitus at an endocrinology reference center. Before her diagnosis, she did not perceive alterations in the redistribution of body fat since her parents also presented a similar phenotype. She also had diabetes mellitus, hepatic steatosis, and severe hypertriglyceridemia (up to 4.814 mg/dL) but no prior pancreatitis. Fenofibrate 250 mg/day was started, reducing triglyceride levels to 1.885 mg/dL. Despite high-dose NPH insulin, regular insulin, and metformin, she maintains unsatisfactory glycemic control (HbA1c 10,7%). Phenotypic changes included reduced adipose tissue in the upper and lower limbs, phlebomegaly, and large numbers of eruptive xanthomas affecting the elbows, buttocks, and posterior region of the thigh since she was 14 years old (Fig. 1A). She had non-consanguineous parents, and her three sisters and five brothers presented similar phenotypic features and a history of diabetes mellitus, dyslipidemia, and pancreatitis. Her family members did not consent to the genetic screening, and the patient had difficulty adhering to the treatment. Her biochemical results are reported in Table 1.

**Table 1:** Biochemical findings and clinical evaluation of the FPLD3 patients

	Case 1	Case 2	Case 3
Pathogenic variant at cDNA level	c.533 T > C	c.641 C > T	c.641 C > T
Model of Inheritance	AD	AD	AD
Pathogenic variant at the protein level	p.(Leu178Pro)	p.(Pro214Leu)	p.(Pro214Leu)
Sex	Female	Female	Male
Age (years)	38	34	32
Weight (kg)	58	64.4	74.3
Height (m^2^)	1.60	1.66	1.66
BMI (kg/m²)	22.6	23.1	26.7
Fat body mass %	NA	29.3	24.6
Upper limb fat %	NA	30.7	22.2
Lower limb fat %	NA	20.5	17.6
Trunk fat %	NA	34.9	30.5
Fat mass ratio (FMR)	NA	1.70	1.73
Android to gynoid fat ratio (AGR)	NA	1.56	1.6
Baumgartner’s index (ASM/height^2^)	NA	6.75	9.3
Fasting glucose (mg/dL)	252	86	96
Triglyceride (mg/dL)	1.885	347	448
HDL Cholesterol (mg/dL)	24	10	33
LDL Cholesterol (mg/dL)	170	99	127
Total Cholesterol (mg/dL)	406	232	179
Insulin (µU/mL)	30.1	29.2	75.5
HbA1c %	10.7	NA	5.6
AST U/L	35	25	44
ALT U/L	37	22	25
Leptin ng/mL*	0.8	0.85	1.05
Adiponectin µg/mL**	2.2	1.5	1.1

All leptin and adiponectin reference values follow the parameters of the Brazilian Society of Cardiology, Diabetes, and Obesity (7,8)

* For the diagnosis of lipodystrophy, the leptin values ​​considered are less than 8 ng/mL for males or less than 12 for females (25)

**Adiponectin values for all FPLD3 subjects described here were similar to those of previous data (47)

The fat mass ratio (FMR) was calculated by dividing trunk fat % by lower limb fat %

Baumgartner’s index was calculated by dividing appendicular skeletal muscle by height^2^ (ASM/height^2^) (10)

NA denotes not available

AD denotes autosomal dominant

Parentheses were included in the pathogenic variant at the protein level since no experimental data was obtained, and the protein change predicted by NGS was confirmed using Mutalyzer and Mutation Taster tools (15,18)

#### Case 2

An 8-month pregnant 34-year-old female patient (sibling of case 3) with dyslipidemia was clinically evaluated at an endocrinology reference center. She was diagnosed with high levels of triglycerides at age 28, with no prior history of diabetes, hypertension, or atherosclerotic disease. She has previous reports of triglycerides reaching 2.144 mg/dL and steatosis and pancreatitis. Her menarche was at age 12, with regular cycles without hirsutism. She has twin daughters. She presented reduced adipose tissue in the upper and lower limbs (Fig. 1B). During the clinical evaluation, she used fibrate and omega-3 without satisfactory results. After the clinical and biochemical assessment, the occurrence of steatosis and pancreatitis was noticed. Her biochemical results are described in Table 1.

#### Case 3

A 32-year-old male patient referred by his sibling (case 2) was clinically evaluated at an endocrinology reference center. He was diagnosed with dyslipidemia (hypertriglyceridemia and low HDL) with no prior history of diabetes, hypertension, steatosis, atherosclerotic disease, or pancreatitis. He did not have phenotypic and clinical features of partial lipodystrophy (Fig. 1C). His biochemical results are described in Table 1.

### Molecular and Bioinformatics Analysis

**Fig. 2:**
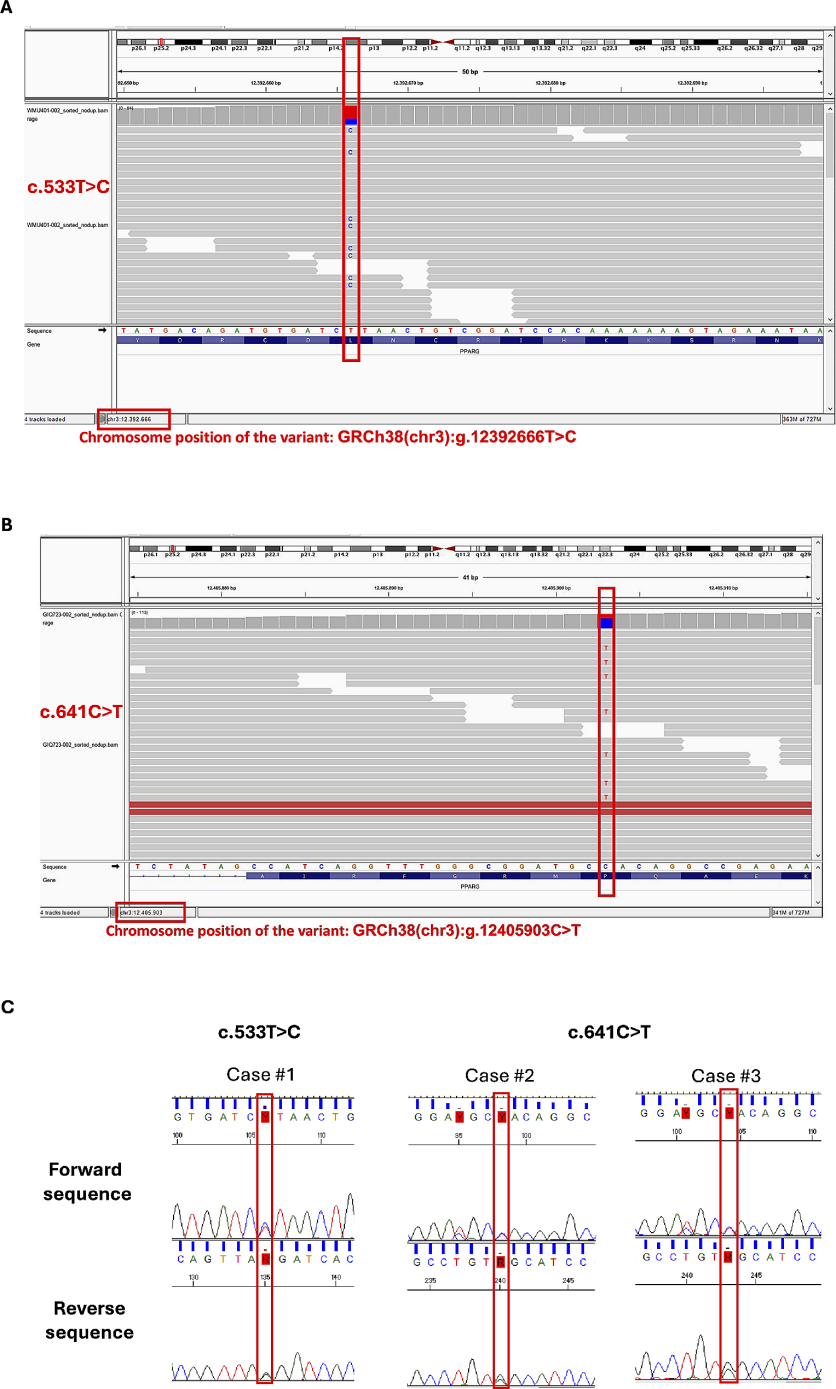
NGS and Capillary DNA sequencing of the new variants in the *PPARG* gene. (**A**) and (**B**) NGS sequencing results for the c.533T > C and c.641 C > T variants, respectively. In both cases, the mutated nucleotide is highlighted with a red box. The chromosome position is also provided. (**C**) Capillary DNA sequencing results reveal the heterozygous variant in forward and reverse sequences. Y represents the thymine or cytosine nucleotides corresponding to the variant found, and R represents the adenine or guanine nucleotides. Variant annotation was based on sequence NM_015869.5

NGS and capillary DNA sequencing revealed that the three clinically diagnosed FPLD patients had pathogenic variants in the *PPARG* gene (Fig. 2). Case 1 presented the c.533T > C (NC_000003.12:g.12392666T > C) variant affecting the DBD domain of the PPARγ protein. Cases 2 and 3 had the c.641 C > T (NC_000003.12:g.12,405,903 C > T) variant affecting the Hinge domain. Both variants were named according to HGVS recommendations [14], deleteriousness predictions were obtained according to ACMG (Table S2), and submitted to the Clinvar database, a public archive of *homo sapiens* genetic variants and their relationships to the clinical features of diseases [23]. The registration numbers for the novel *PPARG* variants in the Clinvar database are SCV004697857 (c.533T > C) and SCV004697856 (c.641 C > T). As recommended by ACMG, we applied in silico predictive algorithms to predict the impact of both c.533T > C and c.641 C > T *PPARG* missense variants. PolyPhen-2 deleteriousness predictions revealed that both are probably pathogenic. Mutation Taster, CADD, and REVEL predictions confirmed the deleteriousness of both variants (Table S2). All ACMG criteria of both missense variants are described in Table S2.

It is worth noticing that although the c.641 C > T has a rs number (rs2050642774) and was previously found in two FPLD3 subjects by Garg et al. in 2022, they neither provided a detailed case description nor a detailed analysis of this *PPARG* pathogenic variant. They inserted data from these subjects with data from all FPLD3 cases to compare the biochemical and phenotypic differences between FPLD3 and FPLD2 groups [24]. Before our work, the novel two heterozygous *PPARG* pathogenic variants described here were not registered, and no phenotype-genotype relationship was provided in the scientific literature. Here, we provided a phenotype-genotype association of both novel *PPARG* pathogenic variants affecting the DBD domain and the Hinge region and compared it with previous variants affecting these PPARγ regions (Table 2).

**Table 2:** Genotype-phenotype association of the most frequent *PPARG* variants related to FPLD3

DBD domain															
Pathogenic variant at cDNA level	**Number of subjects (Gender)**	**Pathogenic variant at the protein level**	**BMI** **kg/m²**	**Leptin ng/mL** *	**Adiponectin** **µg/mL** **	**Hypertriglyceridemia**	**Diabetes mellitus 2**	**Acanthosis nigricans**	**Insulin Resistance**	**Hypertension**	**Polycystic ovary**	**Irregular menstrual cycle**	**Esteatosis**	**Eruptive xanthoma**	**Reference**
c.413_416delAATG	1 F	p.(Glu138ValfsX168)	33	NA	NA	+	+	NA	NA	-	NA	NA	NA	NA	Hegele et al. 2006
c.413_416delAATG and c.490 C > T***	1 F	p.(Glu138ValfsX168) and p.(Arg164Trp)	21.2	0.47	1.5	+	+	+	+++	-	NA	NA	+	+	Dyment et al. 2014
c.424T > C	1 F	p.Cys142Arg	30	NA	NA	+++	+	+	+++	+	+	-	+	-	Agostini et al. 2006
c.452 A > G	2 F	p.(Tyr151Cys)	23–24	6.1 (in 1 subject)	NA	+ (in all subjects)	+ (in all subjects)	+ (in 1 subject)	+ (in all subjects)	+ (in all subjects)	+ (in 1 subject)	+ (in 1 subject)	-	-	Visser et al., 2011
c.452 A > G	1 F	p.(Tyr151Cys)	23.5	1.6	NA	+	-	-	+++	-	-	-	-	-	Alvarez et al. 2021
c.476G > A	1 F	p.Cys159Tyr	24.2	NA	NA	+	+	+	+++	+++	+	+	+	-	Agostini et al. 2006
c.482G > T	1 F	p.(Gly161Val)	27	NA	NA	+	+	-	+	+	NA	NA	+++	+	Lau et al. 2015
c.494G > C	2 F	p.Arg165Thr	23.9–28.6	NA	NA	++ (in all subjects)	+ (in all subjects)	+ (in 1 subject)	+ (in all subjects)	+++ (in all subjects)	NA	NA	+ (in all subjects)	NA	Auclair et al. 2013
c.533T > C (Case1)	1 F	p.(Leu178Pro)	22.6	0.8	2.2	+++	+++	-	NA	-	-	-	+++	+++	
c.562 A > C	7 (F), 8 (M)	p. Glu157Asp	NA	F 0.96 M 0.98	F 4.25 M 7.45	+ (in 6 subjects)	+ (in 4 subjects)	+ (in 5 subjects)	- (in all subjects)	+ (in 1 subject)	NA	NA	NA	+ (in 5 subjects)	Campeau et al., 2012
c.568T > A	1 F	p.Cys190Ser	29.8	NA	NA	+	+	+	+	NA	-	-	NA	+	Campeau et al., 2012
c.570T > G	1 F	p.Cys190Trp	30.5	NA	NA	+	+	+	++	++	-	-	NA	NA	Agostini et al., 2006
c.580 C > T	1 F	p.Arg194Trp	25	NA	NA	+++	+	++	+++	+	+	+	NA	+	Monajemi et al. 2007
c.581G > A	1 F	p.Arg194Gln	24	4.3	2.5	+++	+	NA	+	+	+	+	NA	NA	Majithia et al. 2016
NA	1 F	p.Met203Ile	26	5.4	4.0	+++	+	NA	+	-	+	+	NA	NA	Majithia et al. 2016
HINGE region															
c.634 C > T	1 F	p.Arg212Trp	28	5.2	0.8	+	+	NA	+	+	+	+	NA	NA	Majithia et al. 2016
c.635G > A	1 F	p.(Arg212Gln)	NA	5.3	NA	+	+	+	+	+	+	+	+	+	Sorkina et al. 2015
c.641 C > T (Case2)	1 F	p.(Pro214Leu)	23.1	0.85	1.5	+	-	-	NA	-	-	-	+	-	
c.641 C > T (Case3)	1 M	p.(Pro214Leu)	27	1.05	1.1	+	-	-	NA	-	-	-	-	-	

The three new FPLD3 cases described in this research are highlighted in gray

F: Female; M: Male

NA denotes not available

(-) denotes the absence of the clinical sign

(+) denotes the presence of the clinical sign and is relative to its severity

All leptin and adiponectin reference values follow the parameters of the Brazilian Society of Cardiology, Diabetes, and Obesity (7,8)

* Reference values for leptin: BMI < 25 kg/m^2^: Male: 0.3–13.4 ng/mL; Female: 4.7–23.7 ng/mL; BMI 25–29.9 kg/m^2^: Male: 0.5–14.6 ng/mL; Female: 4.1–14.5 ng/mL

** Reference values for adiponectin: BMI < 25 kg/m^2^: Male: 4.0–26 µg/mL; Female 5.0–37 µg/mL; BMI 25–29.9 kg/m^2^: Male: 4.0–20 µg/mL; Female 5.0–28 µg/mL

For the diagnosis of lipodystrophy, the leptin values ​​considered are less than 8 ng/mL for males or less than 12 for females (26). Adiponectin values for all FPLD3 subjects described here were similar to those of previous data (47)

*** This subject presents a phenotype of a generalized sWAT loss similar to CGL.

Parentheses were included in pathogenic variants at the protein level with no experimental data to confirm the protein change

**Fig. 3:**
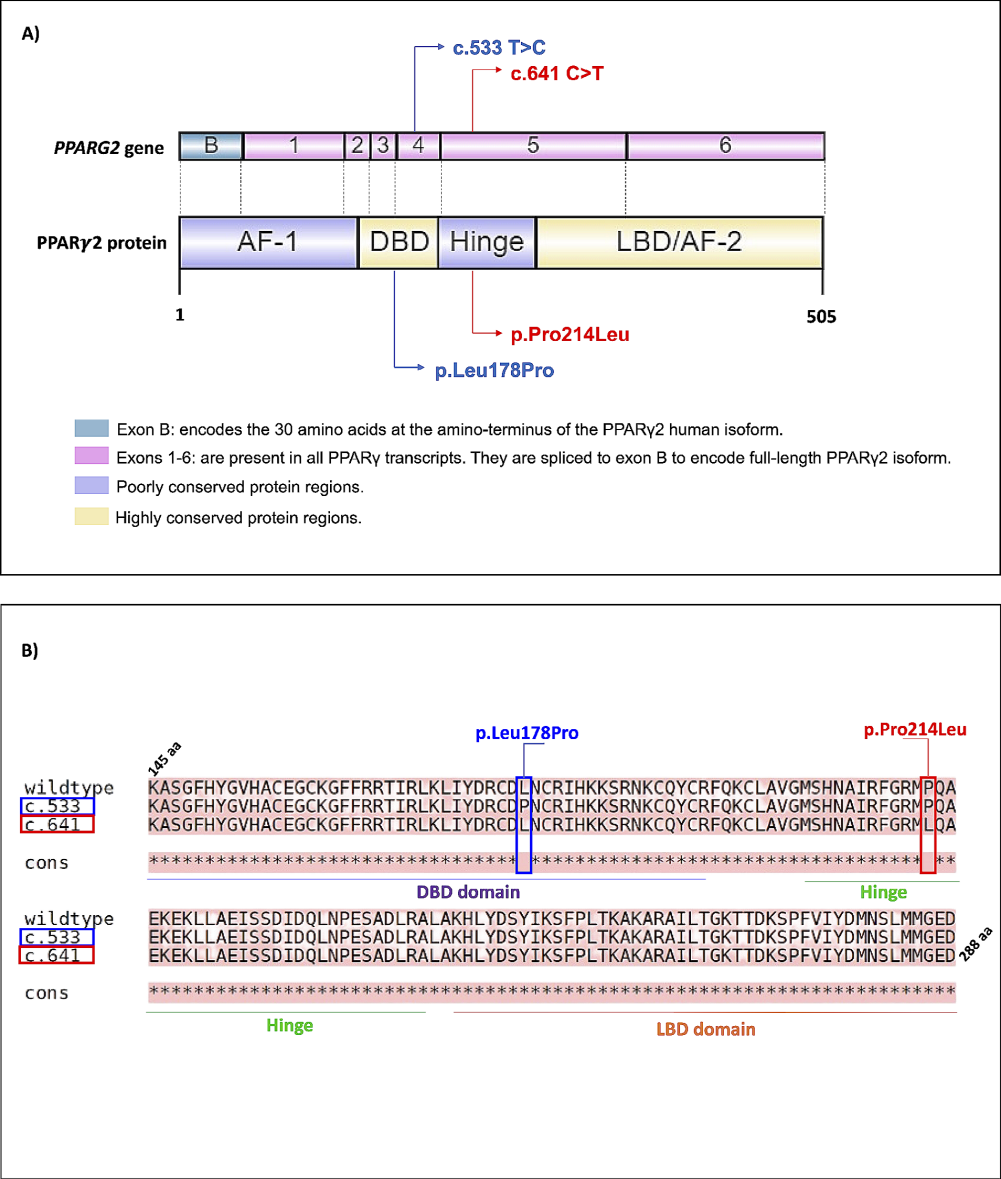
The *PPARG* gene and PPARγ2 protein structure and the alignment of wild-type and mutated PPARγ2 sequences. (**A**) The *PPARG* gene is composed of 7 exons. The positions of the new pathogenic variants are indicated in blue and red. PPARγ2 protein domains are placed according to their exons (not precise in scale). The image was made using IBS 2.0 software [25]. The PPARγ2 protein has five main regions. AF-1 domain and Hinge region are poorly conserved, while DBD, LBD, and AF-2 domains are highly conserved. (**B**) Wild-type and mutated PPARγ2 protein sequences were aligned via T-Coffee. The PPARγ2 sequence used was NP_056953.2. In blue, the mutated sequence for c.533T > C is highlighted, which results in the substitution of the amino acid leucine to proline at position 178, and in red, the c.641 C > T variant, which results in the substitution of the amino acid proline to leucine at position 214. The domains are distinguished by colors: purple corresponds to the DBD domain, green corresponds to the hinge region, and orange corresponds to the LBD domain. Inconsistency between sequences is signaled by the absence of * in the indicated location, where the amino acid is exchanged in the mutated sequences compared to the wild type

**Fig. 4:**
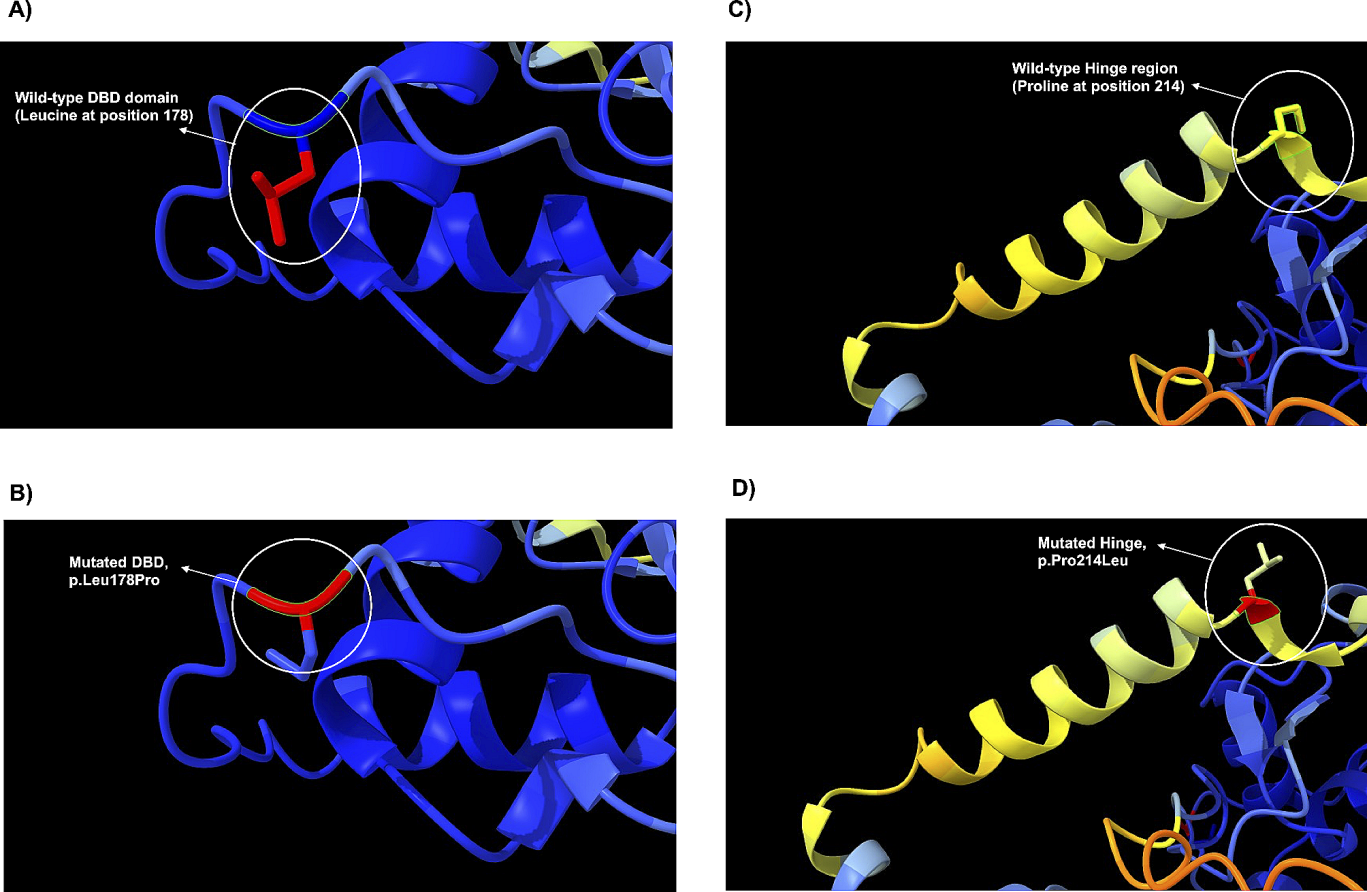
Three-dimensional simulation of PPARγ highlighting the PPARG pathogenic variants. The PPARγ2 sequence used for these analyses was P37231 (NP_056953.2), which comprises isoform 2 with 505 amino acids. In (**A**) wild type and in (**B**) variant region c.533T > C, p.Leu178Pro. (C) wild type and (D) c.641 C > T, p.Pro214Leu.

The amino acids sequence analysis of the wild-type PPARγ isoform 2 (PPARγ2; NP_056953.2) was compared to the mutated PPARγ2 using T-Coffee, which aligned all PPARγ protein sequences and revealed the discrepancies between amino acids sequence (Fig. 3A and B). Since the novel *PPARG* pathogenic variants are missense, the protein sizes are identical with amino acid changes in specific amino acid sites. The c.533T > C variant promotes the substitution of leucine to proline in position 178 (p.Leu178Pro), and the c.641 C > T variant results in the substitution of proline to leucine in position 214 (p.Pro214Leu) at the PPARγ2 protein (Fig. 3A and B). To assess the consequences of these variants on the tridimensional structure of the PPARγ2 protein, we conducted bioinformatics predictions using the AlphaFold tool [22]. We obtained the crystal structure of the wild-type and the mutated (p.Leu178Pro) and (p.Pro214Leu) PPARγ2 proteins. Then, the optimal side-chain rotamer conformation was examined. The results revealed changes in the rotamer conformation of both mutated PPARγ2 proteins compared to the wild-type (Fig. 4). These results suggest that the c.533T > C and c.641 C > T variants can affect the interactions of the PPARγ2 DBD domain with its ligands and impair Hinge functions, respectively.

### Adiponectin and leptin analysis

Since PPARγ upregulates adiponectin to increase insulin sensitivity [26], adiponectin and leptin levels in FPLD3 subjects reviewed in Table 2 were plotted to understand better the relationship among the extension of sWAT loss, the levels of those adipokines, and the PPARγ affected domains (Figure S1). Although adiponectin data is missing for some pathogenic variants (DBD: p.Tyr151Cys; Hinge: p.Arg212Gln), it is possible to verify some variation in its levels even in pathogenic variants affecting the same domain. Similar results were observed for leptin levels, though the majority of FPLD3 subjects presented leptin levels lower than 8 ng/mL (men) and 14 ng/mL (women), as expected for lipodystrophic subjects [27, 28]. The new FPLD3 subjects described here presented similar leptin levels, while adiponectin levels were slightly lower in FPLD3 subjects harboring the Hinge variants (cases 2 and 3) (Figure S1). These data ratify the complexity related to the adipose tissue loss heterogeneity and the expression pattern of adipokines.

## Discussion

The discovery of PPARs in the 1990s was a significant breakthrough in unraveling the adipogenesis pathway and insulin regulation, with peroxisome proliferator-activated receptor gamma emerging as a crucial regulator [29]. In the mid-2000s, researchers found a correlation between the *PPARG* gene and FPLD3. These findings encouraged research concerning the effect of *PPARG* pathogenic variants and the molecular mechanisms related to the FPLD3 phenotype features and clinical comorbidities [6].

Recent studies reveal that partial lipodystrophy can develop from different genetic variants at the same gene or distinct genes. Each subtype of FPLD may exhibit distinct clinical characteristics, and the same FPLD subtype can also reveal a phenotypic heterogeneity, ratifying the complexity of congenital lipodystrophies. FPLD3 subjects may encounter metabolic challenges, even if their adipose tissue loss is not apparent during a clinical evaluation, as observed for other FPLD subtypes [30, 31]. *PPARG*-related lipodystrophy is higher among females after puberty, which may have early menarche and polycystic ovary syndrome [32]. FPLD3 individuals typically display a mild loss of adipose tissue in several body areas. Moreover, there is no correlation between the loss of sWAT and metabolic disorders such as type 2 diabetes, insulin resistance, hypertriglyceridemia, and liver dysfunction [33].

The human *PPARG* gene exhibits some distinctions from that of other species. Nevertheless, the regulatory regions of this gene remain remarkably conserved, as they are crucial for maintaining the cellular functions related mainly to adipogenesis. Conversely, the binding regions may vary across different species [34]. The novel *PPARG* pathogenic variants described here affect the DBD domain and the Hinge region of the PPARγ protein (Fig. 3A and B). Consistent with previous research, the DBD domain appears more susceptible to variation [35]. However, variations in the Hinge region, while less frequent, have also been associated with metabolic effects similar to those observed in other domains [36, 37].

The DBD domain is highly conserved and has a vital role in PPARγ’s function [38]. Variant pathogenic variants in the human PPARγ DBD domain can inhibit its transcriptional roles and lead to severe insulin resistance and increased diabetes risk. The DBD is connected to the LBD domain via a flexible Hinge region, which physically interacts with the DNA [39]. The N-terminal AF-1 domain regulates the transcriptional activity of PPARγ in a ligand-independent manner. The Hinge region interacts with PPAR coactivators and co-repressors, and the LBD domain, along with the C-terminal AF-2 domain, regulates the ligand-dependent transcriptional activity of PPARγ [40–42].

The novel c.533T > C (p.Leu178Pro) *PPARG* variant affects the DBD domain in the PPARγ2 protein, probably hindering its ability to bind DNA and activate transcription of its target genes. Figure 4 shows that this missense *PPARG* variant can result in structural changes in the PPARγ2 protein, which suggests that this variant may decrease the ability of the PPARγ2 protein to interact with its ligands. Considering other *PPARG* variant cases in the literature, it was described that the substitution of arginine to tryptophan (p.R194W) in the DBD domain of PPARγ2 disrupts DNA binding and transcriptional activities, leading to FPLD3, as evidenced by a case report of Monajemi and authors [43]. This research also investigated a nonsense *PPARG* pathogenic variant referred to as p.Y355X, which affects the PPARγ protein structure and disrupts the regulation of its partners. This nonsense variant leads to haploinsufficiency, a reduced amount of functional PPARγ protein, in FPLD3-affected individuals. The p.Y355X variant produces a truncated PPARγ protein with no transcriptional activity, thus affecting fat cell metabolism, demonstrating the mechanism by which the *PPARG* variants lead to FPLD3 [44, 45]. Another study conducted by Chen and co-workers evaluated the conformational changes associated with the p.F310S variant using AlphaFold. They observed that this variant causes a change in hydrogen bond networks at amino acid position 310. This change may affect the network of interactions required to maintain the LBD domain in its active conformation, which may be related to a decreased function of the PPARγ protein [46]. AlphaFold analysis revealed significant conformational changes due to the novel variants (p.Leu178Pro) (DBD domain) and (p.Pro214Leu) (Hinge region) in the PPARγ2 protein. Although there are few reports of FPLD3 cases related to pathogenic variants in the Hinge region [36], the structural amino acid changes due to the (p.Pro214Leu) missense variant probably affect the PPARγ2 interaction with coactivators and the LBD domain. However, more studies must be conducted to understand the molecular mechanisms related to the novel pathogenic variants described here.

Data from previous FPLD3 case reports with pathogenic variants in the DBD domain and Hinge region were also analyzed here, and the genotype-phenotype relationship is provided. We found 31 variants in the DBD domain and 4 cases in the Hinge region in the literature. Among the FPLD3 cases reported in the DBD domain, 61% had type 2 diabetes, 41% acanthosis, 45% insulin resistance, 35% hypertension, 19% polycystic ovary, 16% irregular menstrual cycles, 22% hepatic steatosis, 32% eruptive xanthoma and 70% hypertriglyceridemia. In the Hinge region, 50% had type 2 diabetes, 25% acanthosis, 50% insulin resistance, 50% hypertension, 50% polycystic ovary, 50% irregular menstrual cycles, 50% hepatic steatosis, 25% eruptive xanthoma, and 100% of cases presented hypertriglyceridemia (Table 2). Although less evident, the sWAT loss in FPLD3 individuals is associated with severe metabolic complications described in Table 2, highlighting that the extension of sWAT loss may not determine the severity of partial lipodystrophy per se, as observed in a study that compared FPLD3 and FPLD2 individuals [24]. These findings must be better addressed with a large cohort of partial lipodystrophy related to *PPARG* and *LMNA* genes.

Recent data has demonstrated a clear correlation between altered leptin and adiponectin secretion and the clinical features of congenital lipodystrophy, particularly in cases of generalized sWAT loss, which is more metabolically severe. Lipodystrophic patients commonly experience moderate to severe insulin resistance, as leptin and adiponectin are crucial in insulin sensitization [47]. Here, we found low adiponectin levels in all FPLD3 subjects, which can be related to the metabolic changes associated with the loss of sWAT, mainly in the upper and lower limbs. This finding was also found even in the FPLD3 male (case 3) with no evident loss of sWAT. Previous data from the literature showed that female FPLD individuals tend to have lower adiponectin levels than their male counterparts [48]. This disparity may help explain the higher prevalence of clinical lipodystrophy cases diagnosed in female subjects and the heterogeneity of sWAT loss between genders. Further, FPLD females experience metabolic impairment and other related symptoms, such as polycystic ovary syndrome and irregular menstrual cycles, which may lead them to seek medical attention more frequently. Conversely, many male FPLD patients may not receive adequate diagnosis or treatment for these conditions, like our case 3 patient. Phenotypic changes in male FPLD individuals seem much less prominent than in females, which may imply underdiagnosis [48].

Concerning the leptin and adiponectin levels in the three new FPLD3 cases reported here, we found they also exhibited low leptin and adiponectin levels as all FPLD3 cases reviewed here (Table 2; Figure S1). As previously found by Garg et al. 2002, FPLD2 subjects also presented lower leptin and adiponectin levels. Furthermore, while leptin levels in diabetic and non-diabetic individuals were similar, adiponectin levels were still lower in diabetic subjects than in non-diabetic individuals [48]. Campeau et al., 2012 found that male FPLD3 subjects harboring the c.562 A > C (p.Glu157Asp) in the *PPARG* gene presented low adiponectin (males average 7.45 µg/mL [3.48–8.34]) and leptin (males average 0.98 ng/mL [0.83–1.34]) levels (Reviewed in Table 2) [31]. For the c.641 C > T variant described here, although Garg et al. 2022 found it in two FPLD3 subjects, leptin and adiponectin data for these subjects were not provided [24]. Taken together, our findings are in accordance with previous FPLD3 data, confirming that FPLD subjects display low leptin and adiponectin levels and severe metabolic derangements.

Due to a lack of epidemiological data, it is currently unclear how common FPLD is in Brazil. By describing the variants of this syndrome, we enlarge the FPLD3 epidemiology. It’s worth noting that other types of lipodystrophies, such as CGL, have also been observed in Northeast Brazil [2]. Our data ratify that Brazil has a high prevalence of rare diseases affecting adipose tissue.

Despite the ongoing research on developing new treatments, managing lipodystrophies still primarily focuses on treating the classic symptoms. Standard measures include insulin sensitizers, such as metformin, and lipid-lowering medications, such as statins or fibrates, in cases of severe hypertriglyceridemia. Additionally, the use of metreleptin, a human leptin analog, is part of the therapeutic approach, as it has been shown to improve the metabolic profile, increase satiety, and enhance reproductive function [49].

In conclusion, we described two novel *PPARG* pathogenic variants related to the clinical and phenotypic features of three FPLD3 Brazilian individuals. These and other FPLD case reports in Brazil are crucial to providing new epidemiological data concerning partial lipodystrophies. In addition, these data can encourage more basic and clinical research to enhance the diagnosis and treatment of this underdiagnosed disease in Brazil.

### Electronic supplementary material

Below is the link to the electronic supplementary material.

Supplementary Material 1

Supplementary Material 2

Supplementary Material 3

## Data Availability

Data is provided within the manuscript or supplementary information files.
