# Supplementary figures and images for: Case report: two novel PPARG pathogenic variants associated with type 3 familial partial lipodystrophy in Brazil
Source: Diabetol Metab Syndr. 2024 Jul 1;16:145. doi: 10.1186/s13098-024-01387-9 (PMC11218129; doi:10.1186/s13098-024-01387-9)

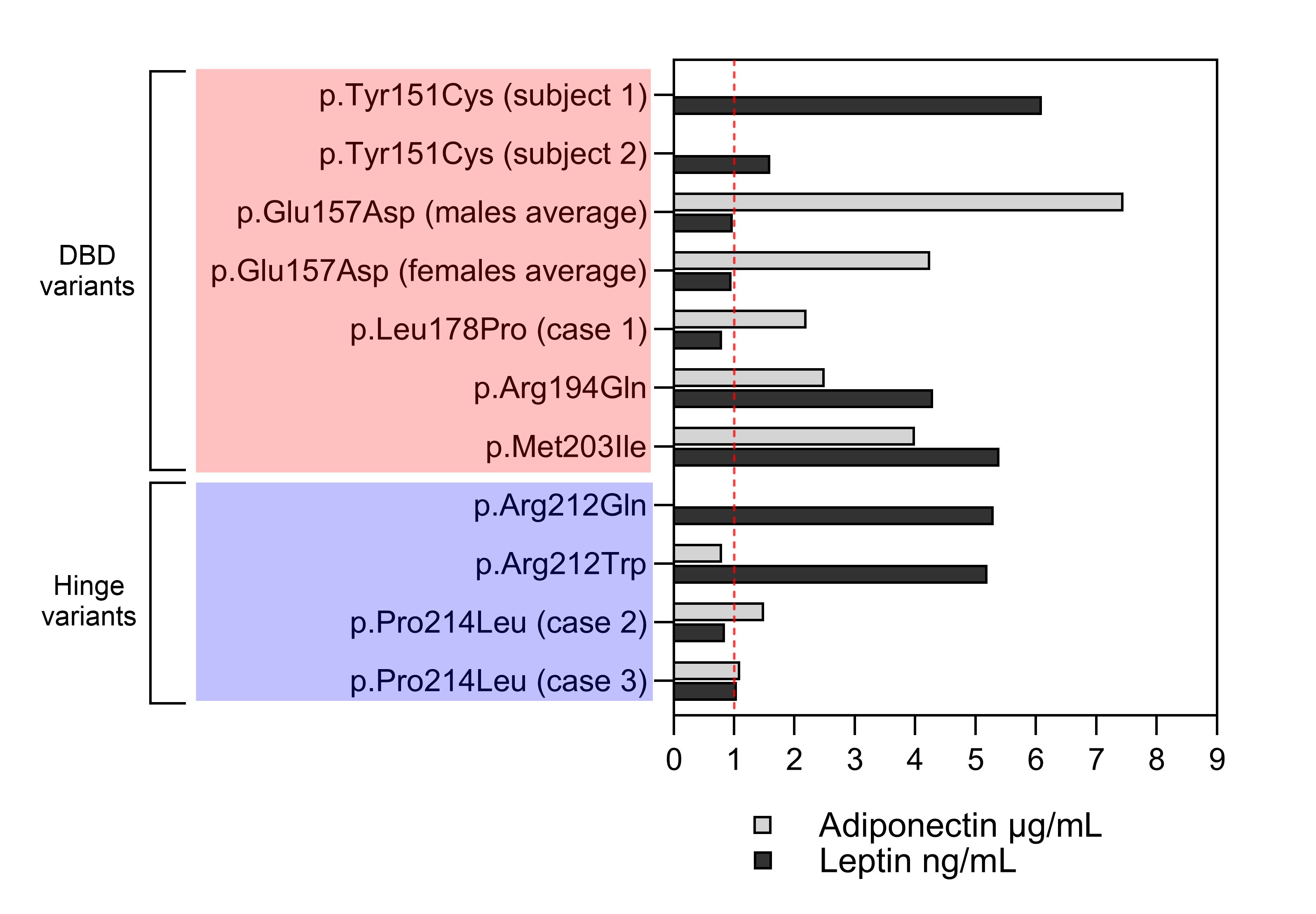

Supplement: Supplementary file 1 — Supplementary Material 1 [file 13098_2024_1387_MOESM1_ESM.png]
