# Supplementary material for: Case report: two novel PPARG pathogenic variants associated with type 3 familial partial lipodystrophy in Brazil
Source: Diabetol Metab Syndr. 2024 Jul 1;16:145. doi: 10.1186/s13098-024-01387-9 (PMC11218129; doi:10.1186/s13098-024-01387-9)
Supplement: Supplementary file 2 — Supplementary Material 2 [file 13098_2024_1387_MOESM2_ESM.docx]

**Case report: Two novel *PPARG* pathogenic variants associated with type 3 familial partial lipodystrophy in Brazil**

| **c.533T>C - GRCh38(chr3):g.12392666T>C (NC_000003.12:g.12392666T>C)** | | | |
| --- | --- | --- | --- |
| **Transcript** | ENST00000397010.7  (NM_001354666.3)  Isoform 1 | ENST00000287820.10 (NM_015869.5)  Isoform 2 | ENST00000397000.6  (NM_001330615.4)  Isoform 3 |
| **Pathogenic variant at cDNA level** | c.443T>C | c.533T>C | c.443T>C |
| **Pathogenic variant at the protein level** | NP_001341595.2:p.(Leu148Pro) | NP_056953.2:p.(Leu178Pro) | NP_001317544.2:p.(Leu148Pro) |
| **c.641C>T -** **GRCh38(chr3):g.12405903C>T (NC_000003.12:g.12405903C>T)** | | | |
| **Transcript** | ENST00000397010.7  (NM_001354666.3)  Isoform 1 | ENST00000287820.10 (NM_015869.5)  Isoform 2 | ENST00000397000.6  (NM_001330615.4)  Isoform 3 |
| **Pathogenic variant at cDNA level** | c.551C>T | c.641C>T | c.551C>T |
| **Pathogenic variant at the protein level** | NP_001341595.2:p.(Pro184Leu) | NP_056953.2:p.(Pro214Leu) | NP_001317544.2:p.(Pro184Leu) |

**Table S1 –** *PPARG* variants found in the new cohort of FPLD3 patients from Brazil.

The transcript used in this research is highlighted in gray (NM_015869.5). The nomenclature of the novel *PPARG* variants at DNA and protein levels was made according to the HGVS standards and guidelines. Parentheses were included in the pathogenic variant at the protein level since no experimental data was obtained, and the protein change predicted by NGS was confirmed using Mutalyzer and Mutation Taster tools (15,18).
