# Supplementary material for: Case report: two novel PPARG pathogenic variants associated with type 3 familial partial lipodystrophy in Brazil
Source: Diabetol Metab Syndr. 2024 Jul 1;16:145. doi: 10.1186/s13098-024-01387-9 (PMC11218129; doi:10.1186/s13098-024-01387-9)
Supplement: Supplementary file 3 — Supplementary Material 3 [file 13098_2024_1387_MOESM3_ESM.docx]

**Case report: Two novel *PPARG* pathogenic variants associated with type 3 familial partial lipodystrophy in Brazil**

**Table S2 –** Deleteriousness predictions of the novel missense *PPARG* variants found in the Brazilian FPLD3 cohort.

| **Tool** | **c.533T>C** | **c.641C>T** | **Range of score** |
| --- | --- | --- | --- |
| **PolyPhen-2** | 0.93 | 1.0 | The score ranges from 0 to 1:  score ≥ 0.7 = probably pathogenic  0.3 ≤ score ≤ 0.6 = intermediate pathogenic potential  0 ≤ score ≤ 0.2 = benign |
| **Mutation Taster** | 98 | 98 | The score ranges from 0 to 215 |
| **CADD** | 24.7 | 27 | The score represents a ranking, not a prediction.  Scores above 20 = variant predicted to be among the 1.0% most deleterious possible changes in the human genome. |
| **REVEL** | 0.73 | 0.71 | The score ranges from 0 to 1  Higher scores = greater likelihood that the variant is disease-causing. |
| **ACMG classification criteria** | PM1, PM2, PP2, PP3, and PP4 | PM1, PM2, PP1, PP2, PP3, PP4, and PP5 | Likely pathogenic:  (v) 2 Moderate (PM1–PM6) and ≥2 supporting (PP1–PP5) |

All in silico predictive algorithms applied here comply with the ACMG standards and guidelines (16).

PM means moderate pathogenicity.

PP means supporting pathogenicity.

PolyPhen-2 (Polymorphism Phenotyping v2) (17).

CADD (Combined Annotation Dependent Depletion) v.1.7 (19).

REVEL (rare exome variant ensemble learner) (20).
